# Implementing a national health research for development platform in a low-income country – a review of Malawi’s Health Research Capacity Strengthening Initiative

**DOI:** 10.1186/s12961-016-0094-3

**Published:** 2016-04-01

**Authors:** Donald C. Cole, Lot Jata Nyirenda, Nadia Fazal, Imelda Bates

**Affiliations:** Dalla Lana School of Public Health, University of Toronto, 155 College Street, Toronto, ON M5T 3M7 Canada; REACH Trust, Health Services Research, PO Box 1597, Lilongwe, Malawi; Dalla Lana School of Public Health, University of Toronto, Health Promotion Evaluation, 155 College Street, Toronto, ON M5T 3M7 Canada; Liverpool School of Tropical Medicine, Pembroke Place, Liverpool, L3 5QA United Kingdom

**Keywords:** Capacity development, Developing countries, Qualitative methods, Research councils, Research management

## Abstract

**Background:**

National health research for development (R4D) platforms in lower income countries (LICs) are few. The Health Research Capacity Strengthening Initiative (HRCSI, 2008–2013) was a national systems-strengthening programme in Malawi involved in national priority setting, decision-making on funding, and health research actor mobilization.

**Methods:**

We adopted a retrospective mixed-methods evaluation approach, starting with information gleaned from reports (HRCSI and Malawian) and databases (HRCSI). A framework of a health research system (actors and components) guided report review and interview guide development. From a list of 173 individuals involved in HRCSI, 30 interviewees were selected within categories of stakeholders. Interviews were conducted face-to-face or via telephone/Skype over 1 month, documented with extensive notes. Analysis of emerging themes was iterative among co-evaluators, with synthesis according to the implementation stage.

**Results:**

Major HRCSI outputs included (1) National research priority-setting: through the production of themed background papers by Malawian health researchers and broad consultation, HRCSI led the development of a National Health Research Agenda (2012–2016), widely regarded as one of HRCSI’s foremost achievements. (2) Institutional research capacity: there was an overwhelming view that HRCSI had produced a step-change in the number of high calibre scientists in Malawi and in fostering research interest among young Malawians, providing support for around 56 MSc and PhD students, and over 400 undergraduate health-related projects. (3) Knowledge sharing: HRCSI supported research dissemination through national and institutional meetings by sponsoring attendance at conferences and through close relationships with individuals in the print media for disseminating information. (4) Sustainability: From 2011–2013, HRCSI significantly improved research systems, processes and leadership in Malawi, but further strengthening was needed for HRCSI to be effectively integrated into government structures and sustained long-term.

Overall, HRCSI carried out many components relevant to a national health research system coordinating platform, and became competent at managing over half of 12 areas of performance for research councils. Debate about its location and challenges to sustainability remain open questions.

**Conclusions:**

More experimentation in the setting-up of national health R4D platforms to promote country ‘ownership’ is needed, accompanied by evaluation processes that facilitate learning and knowledge exchange of better practices among key actors in health R4D systems.

## Background

As part of building national health research capacity [1], both push and pull factors are operating to develop ‘country ownership’. Grappling with the global architecture of aid and the different types of aid disbursed, major international funders have explored options for devolution of research priority setting and funding to low- and middle-income countries (LMICs). Additionally, LMIC governments have increasingly been allocating budgets for planning and commissioning of national health research. These governments and initiatives include middle-income countries like Brazil and India, and lower-income countries (LICs) [2] such as Senegal, which recently created the Fonds d’Impulsion pour la Recherche Scientifique et Technique [3]. Stakeholders from donor countries are increasingly working alongside LMIC colleagues to set research priorities [4], track research funding [2], and promote knowledge translation [5].

For many countries, research capacity strengthening is seen as integral to the development of science, technology and innovation (STI) for broader development purposes, with a range of legal, organizational and operational policy instruments ([6], p. 25). Increasingly, oversight of such functions and policies has been placed with national councils of science and technology, sometimes referred to generically as science granting councils [7]. Core functions of such councils have included research grant disbursements, though many have engaged in a wide range of activities relevant to strengthening capacities of the broader STI system ([7], p. 10). Based on different historical legacies, sectoral mixes, extent of international versus national funding, and approaches to governance and innovation, the organizational structures and mandates of these councils can differ dramatically [7].

As platforms for implementing research for development (R4D), such councils and their programs have faced challenges. Some challenges are inherent in the development of the broader national STI system [6], as exemplified by the experience of health research priority setting in Tanzania: “*As countries increase their investment in research, it is essential to increase investment in research management and governance as well, a key and much needed capacity for countries to make proper use of research investments*” [8]. Other challenges are more relevant to national health research system development efforts which must grapple with mapping existing health research capacity, aligning research allocations from multiple sources with national health needs, and balancing technology development versus health services research [9]. A consultation among Western Pacific countries and their research funders brought forth “*the need to invest more in essential health research and management functions, including establishing publicly accessible web-based national health research registries for prospective registration of health research, setting up systems to archive and share health research data, and improving the governance of research ethics committees*” [10].

This paper is a case study describing one science granting council which operated as a platform for prioritizing, financing and managing health research and development – the Malawi Health Research Capacity Strengthening Initiative (HRCSI). The conception of HRCSI began in 2005 with a scoping and design mission led by three agencies: the United Kingdom Department for International Development, the Wellcome Trust, and the Canadian International Development Research Centre. The mission found that few Malawian institutions were performing research, and research which was being performed was mostly through externally funded individual fellowships or northern institution-led consortia; there was no cohesive national approach to health research. In 2006, the three agencies approached the Government of Malawi to set up a Task Force to develop a proposal for the establishment of a Malawian organization to focus on national research funding. A group of ‘elders’ identified 12 people, representing government, research institutions and civil society, to form the Task Force. Following a broad and inclusive consultation process within Malawi, and with counterparts from a sister project in Kenya, a proposal was submitted to the funders in 2007 (see key events in Table 1).^1^ A funders’ steering group was established and HRCSI incubation started (Box 1). As the National Commission on Science and Technology (NCST) was just being established and did not have the capacity to host the HRCSI, a Malawi-based organisation, Liverpool Associates in Tropical Health Malawian subsidiary, LATH UMOYO, was appointed to manage a 2-year incubation period, during which the systems required for grant making could be set up and the awarding of grants initiated.

**Table 1:** Key events in development and implementation of the Malawian Health Research Capacity Strengthening Initiative (HRCSI)

Year	2005	2006	2007	2008	2009	2010	2011	2012	2013	2014
Events	Funders gauge interest and feasibility	Malawian Task Force consults and develops proposal	Initial proposal to funders	HRCSI starts incubation with Steering Group (funders) and initial project implementers (LATH UMOYO)	Amended version of HRCSI proposal funded and initiation of granting	NCST established HRCSI restructured, secretariat under NCST Board	Extra support and accountability mechanisms introduced for financial and project management; proactive media engagement initiated	National Health Research Agenda promulgated	HRCSI developed guidelines for grant schemes National meeting with presentations by grantees	Consolidation phase ends and external funding ceased
Grants awarded					√	√		√	√	

LATH UMOYO, Liverpool Associates in Tropical Health Malawian subsidiary; NCST, Malawi National Council of Science and Technology

**Table:** 

**Box 1. Malawi**’**s National Commission for Science and Technology** (**NCST**) **and the Health Research Capacity Strengthening Initiative** (**HRCSI**) The NCST was included in the Science and Technology Act (2003) and founded with a Cabinet directive (2008) as a parastatal organization of the Government of Malawi based in Lilongwe. NCST’s purpose is to provide science and technology to the government and to address the existing fragmentation of research efforts and research knowledge across the country in order to accelerate the socioeconomic development of the nation and to improve the quality of life of its people. The NCST Board includes a representative from the Ministry of Health. HRCSI was a 5-year (2008–2013) programme, which aimed to achieve “*strengthened health research capacity for the generation of scientific knowledge and improve its use in evidence-based decision making, policy formulation and implementation.*” HRCSI was a £10,000,000 programme, jointly, and approximately equally, funded by the Wellcome Trust and the United Kingdom Department for International Development. HRCSI’s goal, as set out on the NCST website (http://www.ncst.mw/?s=hrcsi&x=0&y=0), was to “*strengthen national environment for generation of multi-disciplinary research and its uptake to inform policies and interventions…and health of individuals*.” Its expected outputs were: • Improved regulation and co-ordination of national health research • Enhanced institutional capacity for high-quality multi-disciplinary health-related research studies • Effective sharing of scientific knowledge • Evidence-based policy and programme formulation	

The NCST commissioned the authors to conduct a review of HRCSI implementation [11] with the purpose “*to document the performance and impact of HRCSI, and to note successes, challenges and lessons learnt in order to inform future health research capacity strengthening activities in Malawi and in other contexts*.” Given the limited evidence on appropriate ways to review national health research for development (R4D) platforms and the dearth of published evaluation tools, we treated this review as research. This paper describes the instrument development, interviewee selection, data collection and qualitative analysis methods. It shares key results, discusses these in light of other LIC science research council efforts and suggests lessons for the implementation of health R4D platforms.

## Methods

### Design

This is a retrospective mixed methods review.

### Documentation collection

In addition to material available on websites, HRCSI staff provided any versions of proposals, reports on the national research agenda, annual monitoring and evaluation reports by HRCSI, prior independent evaluations, and programs of dissemination conferences organized by HRCSI (*n* = 21). Further, they shared with the review team data files of all project topics and awardees, both individual and institutional, throughout HRCSI’s granting history (2009–2013).

### Framework of key actors and components

**Fig 1:**
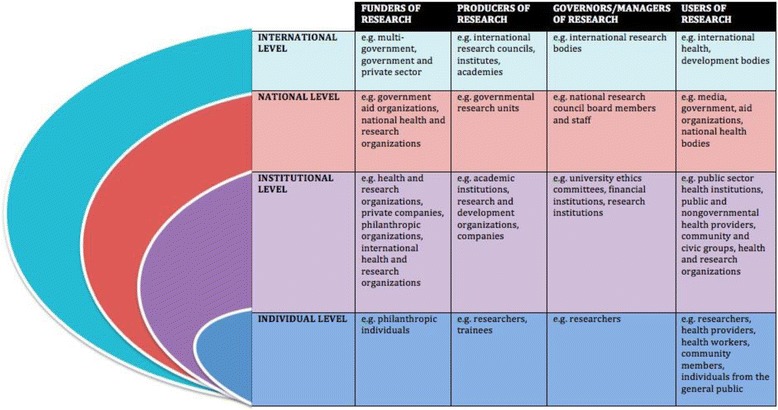
Key actors in an optimal health research system*. *Excluding consultants that cut across all levels of actors

The review framework was grounded in a theoretical understanding of the key actors (equivalent to stakeholders and consistent with social actor theory) and components in an optimal health research system, derived from relevant academic and grey literature [1, 12–14]. We recognized that actors would vary by the level at which they primarily worked, i.e. individual, institutional, national, and/or international levels (see nested ovals in Fig. 1) and the kind of actor they were, i.e. funder, managers, producers or users of research. Knowledge-sharing actors (those who mediate between the funders, managers, producers and users of research) and improvement/management consultants were understood to act across all of the levels (not shown in figure for simplicity). For actors at each level, we set out key components (Box 2) to orient the document review and interview guide development.

**Table:** 

**Box 2. Key components for actors at each level of an optimal health research system International** Collaboration, international research networks, external funding, and information sharing/knowledge exchange **National** Demand for health research, domestic funding, coordination, research networks feedback and evaluation, mission-driven research production, information sharing/knowledge exchange, research use **Institutional** Critical mass of researchers and staff, leadership, infrastructure, funding collaboration, research ethics research management, feedback and evaluation, and information/knowledge sharing **Individual** Human resource development, long-term retention, research use and information/knowledge sharing	

### Interview guide development

Review of the collected documentation and the personal experiences of two members of the review team through prior work and research with Malawian colleagues helped ground the team’s understanding of the health research system in the context of health research and development in Malawi. The full suite of actors and levels were compressed to five different categories for interview guide development: international funders, national governors-managers, national users, institutional/individual producers, and consultants.

Aspects for questions on each of the components, i.e. processes, progress, strengths/assets, impact and lessons learned, were informed by available literature [14]. Different aspects were prioritized in interview guides for different actors. Finally, previously designed tools to evaluate institutional research capacity [15, 16] influenced the structure of the interview guides and provided additional questions.

### Interviews

The HRCSI secretariat provided a list of 173 individuals who had knowledge of the HRCSI programme. In each of the five categories, one-fifth of individuals were initially randomly selected and contacted to arrange an interview; none outrightly refused. If they were unavailable, the next person on the list in that category was approached, so that at least one person in each category was interviewed. Additional interviewees were purposively selected because of their in-depth knowledge in areas or phases of HRCSI development and implementation not sufficiently covered by interviews to date. Of the 30 interviews completed, eight were women and 22 men, with ages ranging from 20–69. Three were international funders, eight were involved in national research governance-management, three were national research users, 14 were producers of research (five institutional research leaders, four researchers and five research trainees), and two were consultants.

Prior to the interviews, the interviewers (usually two people) agreed among themselves on the topics on which each particular interviewee was likely to be able to provide particular insights. The vast majority (90%) of interviewees gave permission for interviews to be recorded as back-up to written notes taken by members of the interview team. Discussions took place between interviewers immediately after each interview, with the goal of corroborating interpretations.

### Analysis

Preliminary analysis by the interview team was focused on major HRCSI outputs and occurred concurrently with report writing in the field (September–October 2014). During this process, the saliency of project phase, i.e. start-up, re-organization, implementation and planning for sustainability, became apparent. Secondary analysis of the data and double-checking of findings were undertaken by non-field team members, using field notes and available interview recordings. Whenever possible, we triangulated interview responses with documentation across interviewees and/or across team members to improve validity of the results. We sought both commonalities and areas of difference across actor groups, and the consequences of differences. We compiled suggestions by interviewees for mitigation of such consequences and synthesized these with our own analysis based on experience and relevant literature to produce lessons for such R4D platforms in the future.

## Results

### Start-up

The start of HRCSI coincided with the formation of the NCST. In the longer term, it was planned that HRCSI would be managed within NCST, but initially NCST had no operational budget or financial operating systems and the funders’ start-up institutional review had not included an in-depth assessment of the capacity in NCST to utilise the HRCSI project budget. One funder wondered whether there “*should have been more time spent on learning by doing instead of on explaining why targets had not been met*”. Funders previous experience of supporting research centres in Kenya and Malawi was not directly applicable to the national level HRCSI. Coming to grips with the extent of the deficit in existing research systems and financial management capacity meant that attention expanded from technical issues to governance mechanisms. As one governor said, “*No one knew their roles and responsibilities*”. Start-up was fraught with a number of difficulties (Table 2): overambitious output targets, poor communication among agencies, budget re-profiling, continued lack of consolidation of skills and structures in NCST, the loss of key LATH UMOYO staff, jockeying among government department personnel regarding the location of HRCSI, and large salary differentials between NCST and HRCSI staff. From the perspective of funding applicants in Malawi, the weak grant-making and communications processes meant that applicants often experienced delays of over 1 year between being selected for an award and the funds being available. Coupled with the lack of clarity about standards against which applications would be judged, the decision to cancel the third round in 2011 after applications had been submitted meant that “*HRCSI lost credibility with stakeholders*” (manager).

### Reorganization

HRCSI was reorganised in 2010–2011. Systems and processes for soliciting and reviewing proposals, for disbursing funds and receiving reports from awardees were strengthened and oversight for the project was made the responsibility of the NCST Board. HRCSI drew upon expertise and lessons from around the region to reform its operations. Examples include multinational research review panels based on experiences from Kenya, design of the national research agenda and knowledge translation processes from Zambia, set-up of a research registry from Botswana, and advocacy using similarities in the national role of universities with Tanzania. In 2011, a way forward was agreed upon between HRCSI and the funders, mediated by an external consultant, which included reducing the knowledge translation component and recruiting a new project manager with research and management credentials. Contracts were formalised, a bank account was opened and HRCSI was able to demonstrate due financial diligence to the funders. As part of this process, it was agreed that the HRCSI Board should approve the selection of award recipients and financial decisions, but in practice the Board’s decisions also had to be approved by the funders. “*HRCSI money comes from the Wellcome Trust* [partially] *and takes much time*” (Research producer). While it was appreciated that the necessary checks and balances should be in place, this cumbersome multi-step process resulted in significant delays in disbursing award funds.

### Implementation

Following the reorganisation, HRCSI began working on generating its outputs, which included improved research regulation and coordination, enhanced generation of research by institutions, and sharing of new research findings in order to inform decision-making for policies and programmes.

#### Priority setting

HRCSI led the development of a National Health Research Agenda [17], including an up-front set of principles: “*country-drivenness, analytical evidence, stakeholder participation, transparency, iterativeness, and value-drivenness*”. Key activities included establishment of a national task force which identified thematic priority research areas using a Delphi method; selection of consultants who then conducted gap analysis studies (based on literature reviews, key informant interviews and focus group discussions), which were reviewed by priority area sub-groups and then the entire task force; and drafting of the research agenda, commenting by advisors, followed by a national stakeholders’ consultative meeting and finalization. Five key criteria were used to assess research proposals submitted to HRCSI against the research agenda: “*1. Current and potential burden of disease; 2. Feasibility and deliverability of the research …; 3. Expected impact of the research on policy/decision making/system changes…; 4. Effects on equity and social justice…; and 5. Contribution to research capacity strengthening in Malawi*” (MoH, undated). Identified priorities were scored against these criteria (1–5 scale), scores were summed across criteria and the priorities ranked based on the sum.

Many interviewees confirmed that the process of developing the nationally harmonised set of health research priorities was widely known about and supported. A number of interviewees had been involved in the consultation process, including a national research user who said, “*The research agenda process and result was very good. There was a start-up committee of very senior people – powerhouses – and then sub committees of others*”. The 2012–2016 Research Agenda was geared towards obtaining evidence aligned with Malawi’s priority needs for health policy and decision-making. Researchers felt that their choice of research topics generally took account of the Agenda but noted that funders and researchers themselves also influenced research topics and that the Agenda should not interfere with academic freedom.

#### Registry design and national funding

HRCSI secured a grant through the Council on Health Research for Development (COHRED) Botswana to facilitate collaboration between the National AIDS Commission, the Commission on Health Research for Development, and the University of Malawi College of Medicine to develop a health research information system (Rhinno) to register research protocols and ethics submissions. Initial findings indicated a shift from 2004–2007, when only about 40% of research applications for research in Malawi were headed by a Malawian, to 64% following HRCSI implementation. As the registry is not fully operational, there has been no systematic analysis of research to date in relation to the Agenda. HRCSI also lobbied the Government of Malawi to fulfil the requirements of the Abuja declaration and allocate 2% of national funds to the Science and Technology research fund. In part fulfilment of this obligation, district health managers were advised to include research in their annual plans and budgets.

#### Awarding process

Informed by a consultative process, HRCSI offered a wide range of types of awards from travel grants and internships to PhD fellowships and institutional grants.

“*The number of people who have benefitted is high compared to other projects*.” (Funder)

Almost all interviewees liked this award diversity, though some interviewees proposed that, in the future, HRCSI should consider focusing its efforts only on areas that are not well covered by other funders. Short-term grants were viewed primarily as a public relations exercise and raising awareness of research, while longer-term grants were seen as likely to strengthen research capacity.

All applications were reviewed by panels of HRCSI, Malawian and some international members. Initial complaints by applicants about the HRCSI grant awarding process included difficulties in submitting proposals, lack of acknowledgment of receipt and lack of feedback about unsuccessful applications, lack of transparency and/or consistency in eligibility and selection criteria, lack of notification when funds were disbursed to awardees’ accounts, and long delays in getting funds once an award had been made.

“*There were problems getting the award letter and funds with over one year between the two. I had to lose some of my existing team due to lack of continuity*.” (Research producer)

In response to these complaints, from 2012 to 2013, HRCSI developed guidelines for all grant schemes, and produced specific tools for managing various stages of the grant awarding process, including baseline assessment, pre-disbursement, site visits, grantee reports, post award reports and post fellowship reports. Concomitantly, enhanced systems for governance, granting and finance/accounting were developed, as one funder noted,

“*The NCST finance and grants management systems have been strengthened with local and regional assistance*.”

Information and communication technology strengthening included computers and office equipment for the NCST.

“*HRCSI is a very important project, especially for IT infrastructure*.” (Governor)

Manuals were developed for awarding and financing grants. The HRCSI appointed a multi-disciplinary review committee, which gained considerable experience in reviewing and awarding proposals with these new supports. As a result, Malawi’s strengths in biomedical research were extended to more multi-disciplinary approaches, including that for a health and social science network.

“*There are now examples of multi-disciplinary work,… which started from a zero baseline*.” (Governor)

Further, HRCSI supported various international research collaborations.

#### Support to institutions and emerging researchers

Initially, only a few institutions were aware of the HRCSI initiative. From 2011, a pro-active advocacy campaign, including the use of print media for disseminating calls for applications, resulted in a significant boost in enquiries and applications. As one manager noted, “*Non academics are asking when the next HRCSI calls are coming out which shows the demand for research*”. The range of awardees expanded, including smaller institutions, which needed the most support.

“*Funds were made available to institutions and people that would not have been able to access them*.” (Governor)

Researcher interviewees welcomed HRCSI’s efforts to improve institutions’ research support systems but they also noted that most institutions did not have strategic plans for research capacity strengthening.

Interviewees were unanimous in appreciating the boost HRCSI had given to interest in research among young Malawians and in supporting the training of substantial numbers of high calibre scientists, as well as in improving the quality of research results in Malawi. HRCSI supported over 400 undergraduate health-related projects, 56 MSc and PhD students in their research training, and 21 other trainees. Among graduates and others (n = 77), 38 were in the biomedical sciences (clinical chemistry, microbiology, molecular statistics); 20 in public health (epidemiology, demography and informatics), and 19 were in social science (anthropology, economics). From a baseline in 2008 of just a handful of MSc and PhD holders across Malawi, by 2013, the Southern Africa Consortium for Research Excellence (SACORE), the Consortium for Advanced Research Training in Africa (CARTA), the College of Medicine at the University of Malawi, and HRCSI together trained or supported 340 postgraduates.

“*HRCSI has trained high calibre people and young scientists have produced good research*.” (Governor)

Many of the interviewees recognised the need to track these graduates and to make sure that there is a comprehensive national strategy for placing them in appropriate posts and providing them with conducive research environments and career development opportunities.

#### Research dissemination and uptake promotion

There was widespread recognition of the way that HRCSI had supported research dissemination, including through national (e.g. in July 2013) and institutional meetings (e.g. College of Medicine, 2012), sponsorship of an issue of the *Malawi Medical Journal*, and by enabling meetings of special interest groups (e.g. for health and social science, Sep 2013). By late 2013, 50% of grantees had already presented research results in national and international conferences, 50% had submitted their papers for publication to various international journals, and seven had papers published in international journals at the time of our review.

Interviewees from the media did not feel that they had adequate opportunities, and also possibly lacked competence, to re-package research for public consumption. Researchers often did not have time to pursue interactions with policymakers intensively.

“*Researchers are not very good at this. Engagement* [with policymakers] *must be right from the beginning in formulating questions*.” (Research producer)

They welcomed recent efforts by the Ministry of Health (MoH) to improve technical support to make engagement with policymakers more effective.

“*Now we have a national knowledge translation committee and promote communities of practice for each discipline*.” (Governor)

Although HRCSI’s ability to promote the translation of research into knowledge for use by policymakers was hampered by the downgrading of this component during the re-organization phase, it did financially and organizationally support the Knowledge Translation Platform established through the Director of Research at the Malawi MoH with Dignitas International, a Malawi-based NGO, to strengthen the ability of researchers and national policymakers to develop and evaluate policy briefs and systematic reviews and to promote their interactions through an interactive website (http://ktpmalawi.org/).

#### Planning for sustainability

External funding for HRCSI began to be phased out in 2013. One national governor commented that “*Funders have not given us enough time to prove that we can manage this project*” and another that “*Two years is too short to be able to demonstrate impact*”. There was overwhelming support among interviewees for HRCSI to continue as a research management centre.

“*Now HRCSI needs to rationalise its operations, concentrate on research systems to promote a research culture*.” (Research producer)

They supported a mandate to act as the hub for channelling research funding from all health research donors, to coordinate priority setting, and to bring people together to feed knowledge into the ‘policy machinery’. Considerable differences were expressed by interviewees about options for the best institutional location of HRCSI (Table 2). Some thought it should “*meld with existing health efforts*” and be located within the MoH research Unit, though a governor warned that the “*MoH may not prioritise HRCSI activities in the long-term because they have put research money into districts*.” The majority view was that it should be integrated into NCST as the coordinating, multi-sectoral agency for research in Malawi. There was general agreement that the systems, processes (including financial), leadership and the health team within NCST, as well as linkages with the Ministry of Education, would need further strengthening.

**Table 2:** Differences in perspectives among actors, by phase and consequences

(a) Among all key actors			
Phase	Difference		Consequences
Start-up	Location of coordinating health research capacity strengthening within government versus outside and within health versus science & technology versus education		Jockeying for location, delays in directorship
Implementation	Grant calls and processes improved over time versus remained non-transparent and halting in process		Frustrations and delays in implementation
Implementation	Ethics review committees functioning and providing independent review versus compromised with conflicted members and acting as resource generator for institutions		Resentments and labelling of committees as obstacles
(b)Among subsets of actors			
Phase	Differences	Actors concerned	Consequences
Start-up, reorganization and implementation	National politics versus international funder decision-making control	Funders, research governors and users	Stalemates on approvals
Start-up, reorganization	Fiscal mismanagement versus civil service obstacles versus consultants not keeping an eye on the ball	Research producers – institutions, researchers, trainees	Delays in funding flows
Implementation	Extended involvement of other institutions in networks versus competition dominated by those already capable	Research producers – institutions, researchers	Some institutions are keen but not funded, areas remain underdeveloped, broader research culture slower to develop
Implementation	Institutional levies on awards and grants unfair disincentive versus need to fund research management within institutions which primarily rely on student teaching income	Research producers – institutions, researchers	Some researchers take funds to organizations outside academy
Implementation	Researchers not performing sufficient mentorship and training roles versus hard to find time for research with existing teaching loads	Research producers – institutions, trainees	Interns and trainees not receiving adequate mentorship

In early 2014, as part of HRCSI’s consolidation phase, a National Health Science Research Committee was formed under the auspices of the NCST, with a mandate to “*Promote, support, coordinate, and regulate research and development*” in a set of health research fields. It includes ethical review for those researchers in institutions without such capacity. Recently, the first meeting of the Knowledge Translation Platform brought together researchers, experts and policymakers around a policy brief on hypertension. Further, the Social Science for Health network has become more established with a membership and notice board, becoming a focal point for such research (Mathildah Chithila, personal communication). However, the extent to which a secretariat associated with the Committee might perform the fuller suite of functions of an R4D platform remains unclear.

## Discussion

### R4D platform development

The process of setting up a national R4D platform was not without considerable challenges in the start-up period partly related to differences in actors’ perceptions of implementation. An important factor was the nascent state of the Malawian NCST at the initiation of HRCSI funding [6]. Given Malawi’s history of primarily international funding directly to a small number of research centres, without strong engagement in national governance (including priority setting) and management, the initial difficulties were perhaps not surprising, particularly given some of the tensions associated with health research capacity strengthening [18]. In response to a similar situation in the Western Pacific, Rani et al. [10] called for substantial investment in “*essential health research and management functions*”, something which HRCSI did in the NCST. Further, HRCSI confronted the thorny “*research management and governance*” issues which de Haan et al. [8] noted during research priority setting in Tanzania, and which Mills et al. [19] noted during a mid-term review of the HRCSI sister body, the Consortium for National Health Research in Kenya.

In the case of HRCSI, the challenges forced a re-organization and renewed emphasis on research management systems, which eventually bore fruit in sets of guidelines and procedures appropriate to a national R4D platform and enhanced national health research capacity. In terms of our framework of components, HRCSI did stimulate demand for health research, lobbied for domestic funding, engaged in coordination, developed research networks feedback and evaluation, promoted information sharing/knowledge exchange, and contributed to structures to promote research use. In terms of the 12 areas of performance for research councils ([7], p. 38), HRCSI became competent in managing over half: setting research agenda/research priorities, disbursement of research grants (various categories), disbursements of scholarships and loans (mostly masters and doctoral students), capacity-building/training of researchers, funding support for infrastructure development, valorisation of results (dissemination and uptake of research reports and findings), and supporting scientific publishing/scientific journals (n = 7). Such results were consistent with the UNESCO report on Malawi, which cited HRCSI as an example of an achievement in science and technology research funding ([6], p. 93).

### Institutional location of national research councils or R4D platforms

When approaching health research systems as a whole, Ghaffar et al. [12] described a number of different options for the types and locations of coordinating bodies. Focusing on science and technology for innovation councils in sub-Saharan Africa, Mouton et al. [7] discerned different models or cases distinguishing the role of the funder (principal) and the agent (council): (1) the paradigm case in which the government was the primary funder through a national funding council for all sectors, to research organizations; (2) the sector-differentiated model, similar to the paradigm case, but with sector specific councils; (3) the multiple principle-agents model, in which different principals fund through different agents; and (4) the embedded agent case where the council or agent is part of the government. Given the preponderance of international sources of funding in most LICs, the first would be unusual, as multiple principles are common. The Malawian MoH Research Unit might be an example of the embedded model. In terms of agents, HRCSI could be seen to be along the lines of the sector-differentiated model during its tenure, somewhat separate from government and focused on health, although the longer term vision was for it to extend its remit to cover all sectors. However, it does not neatly match any of the models or cases, despite the utility of their exposition and examples from other countries in sub-Saharan Africa by Mouton et al. [7]. Furthermore, the final host for HRCSI was arrived at following intense jockeying among government departments, a scenario applicable across the civil service where projects perceived to have more financial and other benefits are more attractive. Given the perceptions of interviewees and differences over preferred location of a national R4D platform such as HRCSI, some further discussion is likely warranted, as it would be in other contexts where R4D platforms are being established or reviewed.

### Lessons

Some general lessons can be learned from the Malawian experience, some of which are summarized in Table 3. In particular, this case study highlights the importance of adhering to the principals involved in designing capacity strengthening programmes – starting small and expanding gradually, finding and building on what exists already, and establishing trusting and well-defined partnerships [18, 20]. Having a clear sense of the baseline of research council functioning is important, as is the consensus-building role of inclusive priority setting. Key lessons also include making sure that the structures, systems and processes are fit for the purpose of awarding grants before calling for applications, and placing a higher priority on these functions early in the development of a research and development platform. Given the extent to which funding for research and research training in LICs is currently grossly inadequate, including Malawi [21], a variety of mechanisms for research management are likely appropriate. Further, reaching out to a wide range of types, sizes and functions of institutions to engage them in implementation research is appropriate.

**Table 3:** Common perspectives among actors and associated lessons learned, by phase

Phase	Perspective	Lessons
Start-up	Fraught with difficulties, primarily because the incubation phase was overly ambitious and unrealistic assumptions had been made about the existing baseline capacity in Malawi for grant-making	In-depth review of the systems is needed to absorb, disburse and account for the funds and a plan to fill any gaps Sufficient time should be set aside for establishing roles, responsibilities and relationships between all the partners Consider a separate start-up phase (0 to 12–18 months) from a ‘production’ phase (12–18 to 42–48 months), with funding of the second phase contingent on effective systems in place
Reorganization	Extra support and accountability were mechanisms introduced to improve financial and project management	Make sure the structures, systems and processes are fit for the purpose of awarding grants before calling for applications
Implementation	The National Health Research Agenda (2012–2016) developed through background papers and broad consultation was ‘highly commended’ Development of a registry of research to capture protocols and ethics submissions and to track fulfilment of the research agenda remained at an early stage	Consensus building around priority setting is a crucial initial function of an R&D platform
Implementation	Contributed to enhancing mechanisms in Malawi for managing research processes and funding The award process was generally viewed as non-corrupt but consistent reports of problems with the application process remained, including difficulties with submission and poor communication about the outcome of applications HRCSI Developed supporting guidelines and tools for the various stages of the grant awarding process Post award approval, most interviewees were satisfied with their interactions with Health Research Capacity Strengthening Initiative (HRCSI), including professional staff, as well as during organized site visits	Place a higher priority on these functions early on in the development of a research and development platform
Implementation	HRCSI produced a step change in fostering research interest among young Malawians and enlarging the number of high calibre scientists in Malawi; the diversity of awards was popular, with short-term grants raising awareness and providing research exposure, and longer-term grants achieving strengthened capacity to do research	The huge appetite for more training in health research in lower-income countries is currently under-met
Implementation	An advocacy campaign succeeded in making awards to some of the smaller institutions, including those in the non-government sector	Institutions in a range of types, sizes, and functions are able to engage in research
Implementation	Research dissemination occurred through national and institutional meetings and academic media, and by sponsoring attendance at conferences (approximately half of all projects were presented); potential for disseminating research results to the general public through local radio and television media was not fully exploited New initiative with non-governmental organisations was designed to bring together policymakers, subject experts and researchers for the purpose of catalysing research uptake	Knowledge transfer and promotion of research utilization a key function which needs explicit resourcing in R&D platforms
Planning for sustainability	Interviewees voiced overwhelming support for continuation of HRCSI as a national research management centre, with the long-term vision that it could be a national hub for grant management across all sectors (starting with Education), more firmly embedded within National Council of Science and Technology (NCST)	Further strengthening of systems, processes and leadership within NCST and linkages to other sectors needed.

### Limitations

Due to time limits and financial constraints (evaluation budget approximately 0.5% of HRCSI project spend), only about 20% of the total 173 individuals associated with HRCSI could be interviewed. However, none of the interviewees identified any other individuals who were not on the list and whom they thought should be interviewed. Further, as individuals from all five categories were interviewed and saturation points were reached for all the major points, it is unlikely that additional interviews would have resulted in significant new information. However, within the scope of our review, we were not able to undertake in-depth explorations of the major points identified.

Further bibliometric follow-up monitoring and analyses are needed to pick up the long tail of publication and assess its relationship to the National Health Research Agenda and its priorities. Assessment of the longer term impacts of HRCSI and its funded research would take longer but would provide an opportunity for LIC R4D platforms to engage in follow-up evaluation which can support mutual learning [22].

Finally, evidence on the course of R4D platforms in LICs remains limited, making a fuller discussion of the pros and cons of various approaches and the linkages with our framework of actors and components remain rudimentary.

## Conclusions

This work contributes a framework for collecting data on key actors and components in a research system. It considers how a research council or R4D platform’s performance might be analysed in relation to them. Given the paucity of case studies of health R4D platforms in LICs, even while important shifts to LIC ‘ownership’ of research are occurring [23], further case studies and research are warranted to jointly build better evidence and improve R4D platform setup with ongoing quality improvement.

## Abbreviations

HRCSI: Health Research Capacity Strengthening Initiative
LATH UMOYO: Liverpool Associates in Tropical Health, Umoyo
MoH: Ministry of Health
NCST: National Commission for Science and Technology
R4D: Research For Development.

## Footnotes

1 Anon. HRCS Initiative Proposal for Malawi. Amended February 2009.
